# Supplementary material for: Antimicrobial Peptides (AMP) in the Cell-Free Culture Media of Xenorhabdus budapestensis and X. szentirmaii Exert Anti-Protist Activity against Eukaryotic Vertebrate Pathogens including Histomonas meleagridis and Leishmania donovani Species
Source: Antibiotics (Basel). 2023 Sep 19;12(9):1462. doi: 10.3390/antibiotics12091462 (PMC10525888; doi:10.3390/antibiotics12091462)

Supplementary Material S1: Experimental Details

Experimental Details of the *Histomonas* Experiments:

(By Coauthor Petra Ganas)

Experiment 1: Determination of Antagonistic Activities of EPB CFCMs in *Histomonas*/ *Escherichia coli* monoxenic Cultures

Materials and Methods:

The protozoan *Histomonas meleagridis* Turkey/Austria/2922-C6/04 was grown as a monoxenic culture with *E. coli* DH5α in Medium 199 containing Earle's Salts, L-glutamine, 25 mM HEPES and L-amino acids (Invitrogen/GIBCO), 15% heat-inactivated fetal bovine serum FBS (Invitrogen/GIBCO) and 0.22% rice starch for 72 hours at 40°C.

For the preparation of cell-free filtrates 3 falcon tubes containing 9 ml Medium 199 with Earle's Salts, L-glutamine, 25 mM HEPES, and L-amino acids (Invitrogen/GIBCO) and supplemented with 15% heat inactivated fetal bovine serum FBS (Invitrogen/GIBCO) and 0.22% rice starch were inoculated with the bacterial species TT01 yellow or TT01 red and incubated 65 hours at 30 °C in a shaker (225 rpm). Bacterial cultures were centrifuged at 3300xg for 5 min and then the supernatants from the cultures were filtered through 0.22 µm cellulose acetate filters (Millipore).

The bacterial species were grown on MacConkey agar plates before they were transferred to the liquid medium. Two different types of colonies for TT01 were observed on the agar plates: red-brown colored colonies which adsorbed the neutral red from the MacConkey agar, and yellow-colored colonies which did not do so. Both types of colonies were tested for the effect of cell-free filtrates on *H. meleagridis* cells.

The culture medium of the protozoan culture was removed and replaced by the cell-free filtrates of the bacterial cultures. Unchanged protozoan cultures and protozoan cultures in which the culture medium was replaced by fresh Medium 199 supplemented with 15% FBS were used as controls. Each of the different filtrate analyses was performed in triplicate. The cultures were incubated at 40 °C and evaluated every 24 hours by counting the protozoan cells.

At the same time points as the protozoan cells were counted, 50 µl of the cultures were transferred to Coliformen agar plates to evaluate any effect on the bacterial strain *E. coli* DH5α. The agar plates were incubated at 37 °C overnight.

Experiment 2: Determination of Cytopathogenic Effects of EPB CFCMs on Permanent chicken liver (LMH) cells

Permanent chicken liver cells (LMH; ATCC Number: CRL-2117™) were grown in Medium RPMI 1640 (Invitrogen/GIBCO) supplemented with 10% heat-inactivated fetal bovine serum FBS (Invitrogen/GIBCO), penicillin G (200 IU/ml) and streptomycin (200 μg/ml). Cells were inoculated into 25 cm^2 flasks with filtered caps (Sarstedt) containing an end volume of 7 ml culture and incubated in a controlled atmosphere of 5% CO_2 at 37 °C and around 85-90% humidity. After 72 hours of incubation, a confluent monolayer of LMH cells was obtained per flask.

For the preparation of cell-free filtrates 3 falcon tubes containing 9 ml Medium 199 with Earle's Salts, L-glutamine, 25 mM HEPES, and L-amino acids (Invitrogen/GIBCO) and supplemented with 15% heat-inactivated fetal bovine serum FBS (Invitrogen/GIBCO), and 0.22% rice starch were inoculated with the bacterial species EMA, EMC, TT01 yellow or TT01 red and incubated 65 hours at 30 °C in a shaker (225 rpm). Bacterial cultures were centrifuged at 3300xg for 5 min and then the supernatants from the cultures were filtered through 0.22 μm cellulose acetate filters (Millipore).

The bacterial species were grown on MacConkey agar plates before they were transferred to the liquid medium. Two different types of colonies for TT01 were observed on the agar plates: red-brown colored colonies which adsorbed the neutral red from the MacConkey agar, and yellow-colored colonies which did not do so. Both types of colonies were tested for the effect of cell-free filtrates on LMH monolayers.

The culture medium of the LMH monolayers was removed from the flasks and replaced by the cell-free filtrates of the bacterial cultures. Unchanged culture flasks of LMH monolayers and flasks in which the culture medium was replaced by fresh Medium 199 supplemented with 15% FBS were used as controls. Each of the different filtrate analyses was performed in triplicate. The cultures were incubated in a controlled atmosphere of 5% CO_2 at 37 °C, and around 85-90% humidity. Each monolayer was evaluated visually by an inverted light microscope to detect the effect of the cell-free filtrates on LMH monolayers. According to the degree of monolayer destruction, the following scoring system was established: 0=intact monolayer; 1=up to 25% of the monolayer destructed; 2=25-50% of the monolayer destructed; 3=50-75% of the monolayer destructed; 4=more than 75% of the monolayer destructed

Experiment 3: Determination of Cytopathogenic Effects of Serially Diluted EPB CFCMs on Permanent chicken liver (LMH) cells).

Permanent chicken liver cells (LMH; ATCC Number: CRL-2117™) were grown in Medium RPMI 1640 (Invitrogen/GIBCO) supplemented with 10% heat-inactivated fetal bovine serum FBS (Invitrogen/GIBCO), penicillin G (200 IU/ml), and streptomycin (200 μg/ml).

Cells were inoculated into 25 cm^2 flasks with filtered caps (Sarstedt) containing an end volume of 7 ml culture and incubated in a controlled atmosphere of 5% CO_2 at 37 °C, and around 85-90% humidity. After 72 hours of incubation, a confluent monolayer of LMH cells was obtained per flask.

The experiment was performed with the cell-free filtrates EMA 80% and EMC 60%. Bacteria were grown in an LB medium. Eight different concentrations of EMA 80% and EMC 60% were prepared by dilutions with Medium RPMI 1640 (Invitrogen/GIBCO) supplemented with 10% heat-inactivated fetal bovine

serum FBS (Invitrogen/GIBCO), penicillin G (200 IU/ml), and streptomycin (200 μg/ml).

The culture medium of the LMH monolayers was removed from the flasks and replaced by the cell-free filtrates of the bacterial cultures.

Unchanged culture flasks of LMH monolayers and flasks in which the culture medium was replaced by fresh Medium RPMI 1640 supplemented with 10% FBS, penicillin G, and streptomycin were used as controls. Each of the different filtrate analyses was performed in duplicate. The cultures were incubated in a controlled atmosphere of 5% CO_2 at 37 °C, and around 85-90% humidity. Each monolayer was investigated visually by an inverted light microscope to detect the effect of the cell-free filtrates on LMH monolayers. According to the degree of monolayer destruction, the following scoring system was established: 0=intact monolayer: 1=up to 25% of the monolayer destructed: 2=25-50% of the monolayer destructed: 3=50-75% of the monolayer destructed: 4=more than 75% of the monolayer destructed.

RESULTS OF Experiment 1:

**sample**	**filtrate**	**Histomonads * 10^4/ml**									
		**0 h**	**mean**	**24 h**	**mean**	**48 h**	**mean**	**72 h**	**mean**	**96 h**	**mean**
1	TT01 yellow	12,75		20,25		8		4		1,5	
2	TT01 yellow	7,5	9,25	8,75	13,75	2,75	5,083	1,25	2,583	0	0,83
3	TT01 yellow	7,5		12,25		4,5		2,5		1	
4	TT01 red	3,25		2		0		0,25		0	
5	TT01 red	7,5	5,4167	1,75	2,4167	0	0,167	0	0,167	0	0
6	TT01 red	5,5		3,5		0,5		0,25		0	
7	M199+15% FKS	2,5		25,25		57,5		33,75		24,25	
8	M199+15% FKS	3,5	5,583	30,25	32,67	38,25	50,167	15,25	20,67	3	10,167
9	M199+15% FKS	4,75		42,5		54,75		13		3,25	
10	unchange d	6		20,75		26		47,75		57	
11	unchange d	4,5	5,67	18	22,5	27,25	24,67	142,5	74,583	70,5	66,5
12	unchange d	6,5		28,75		20,75		33,5		72	

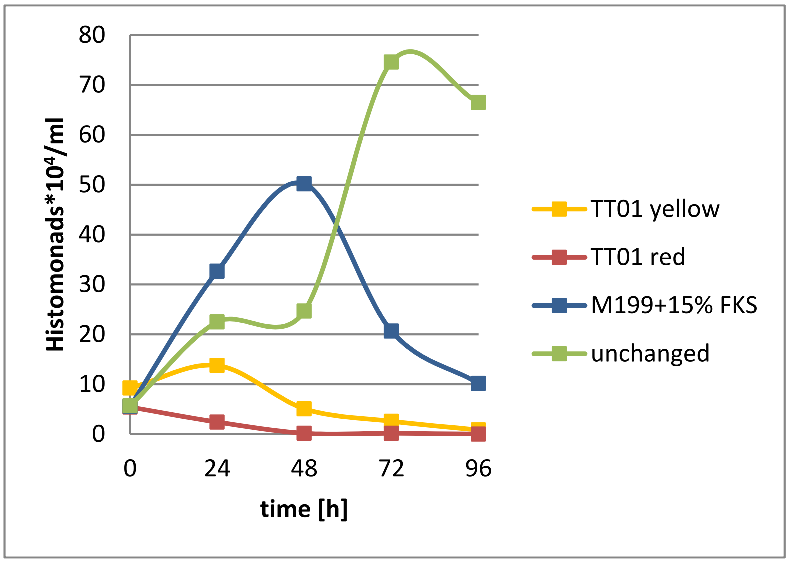

sample	filtrate	*E. coli* DH5α				
		**0 h**	**24 h**	**48 h**	**72 h**	**96 h**
**1**	TT01 yellow	cell layer on whole plate	single colonies on whole plate, but no cell layer more	single colonies on whole plate, but no cell layer more	single colonies on whole plate, but no cell layer more	single colonies on whole plate, but no cell layer more
2	TT01 yellow	cell layer on whole plate				
3	TT01 yellow	cell layer on whole plate				
4	TT01 red	cell layer on whole plate	single colonies on whole plate, but no cell layer more	single colonies on whole plate, but no cell layer more	single colonies on whole plate, but no cell layer more	single colonies on whole plate, but no cell layer more
5	TT01 red	cell layer on whole plate				
6	TT01 red	cell layer on whole plate				
7	M199+15% FKS	cell layer on whole plate	cell layer on whole plate	cell layer on whole plate	cell layer on whole plate	single colonies on whole plate, but no cell layer more
8	M199+15% FKS	cell layer on whole plate	cell layer on whole plate	cell layer on whole plate	cell layer on whole plate	
9	M199+15% FKS	cell layer on whole plate	cell layer on whole plate	cell layer on whole plate	cell layer on whole plate	
10	unchanged	cell layer on whole plate	cell layer on whole plate	cell layer on whole plate	cell layer on whole plate	single colonies on whole plate, but no cell layer more
11	unchanged	cell layer on whole plate	cell layer on whole plate	cell layer on whole plate	cell layer on whole plate	
12	unchanged	cell layer on whole plate	cell layer on whole plate	cell layer on whole plate	cell layer on whole plate	

RESULTS OF Experiment 2:

**0**	intact monolayer of LMH cells
**1**	up to 25% of the monolayer destructed
**2**	25-50% of the monolayer destructed
**3**	50-75% of the monolayer destructed
**4**	more than 75% of the monolayer destructed

**sample**		**24 h**	**48 h**
unchanged	A	0	0
	B	0	0
	C	0	0
M199+15% FKS	A	0	0
	B	0	0
	C	0	0
EMA	A	3	4
	B	3	4
	C	3	4
EMC	A	4	4
	B	4	4
	C	4	4
TT01 yellow	A	4	4
	B	4	4
	C	4	4
TT01 red	A	4	4
	B	4	4
	C	4	4

RESULTS OF Experiment 3:

**0**	intact monolayer of LMH cells
**1**	up to 25% of the monolayer destructed
**2**	25-50% of the monolayer destructed
**3**	50-75% of the monolayer destructed
**4**	more than 75% of the monolayer destructed

**dilution**	**EMA 80%**	**EMC 60%**
1:2.5	32%	24%
1:5	16%	12%
1:7.5	10.63%	7.97%
1:10	8%	6%
1:25	3.2%	2.4%
1:50	1.6%	1.2%
1:75	1.063%	0.797%
1:100	0.8%	0.6%

**sample**		**24 h**	**48 h**	**72 h**	**96 h**
unchanged	A	0	0	0	0
	B	0	0	0	0
RPMI1640+10% FKS+0,5% AB	A	0	0	0	0
	B	0	0	0	0
EMA 80% 1:2.5	A	4	4	4	4
	B	4	4	4	4
EMA 80% 1:5	A	3	4	4	4
	B	3	3	4	4
EMA 80% 1:7.5	A	2	2	4	4
	B	2	2	3	3
EMA 80% 1:10	A	1	2	2	2
	B	1	2	2	2
EMA 80% 1:25	A	1	1	0	0
	B	1	1	0	0
EMA 80% 1:50	A	1	1	0	0
	B	1	1	1	1
EMA 80% 1:75	A	1	1	0	0
	B	1	1	1	0
EMA 80% 1:100	A	1	1	0	0
	B	1	1	1	0
EMC 60% 1:2.5	A	2	3	4	4
	B	2	3	4	4
EMC 60% 1:5	A	1	1	2	2

	B	1	1	2	2
EMC 60% 1:7.5	A	1	1	2	2
	B	1	1	2	2
EMC 60% 1:10	A	1	1	2	2
	B	1	1	2	2
EMC 60% 1:25	A	1	1	0	0
	B	1	1	0	0
EMC 60% 1:50	A	1	1	0	0
	B	1	1	1	0
EMC 60% 1:75	A	1	1	0	0
	B	1	1	0	0
EMC 60% 1:100	A	0	0	0	0
	B	0	0	0	0

**sample**	**mean 0 h**	**mean 24 h**	**mean 48 h**	**mean 72 h**	**mean 96 h**
unchanged	0	0	0	0	0
RPMI1640+10% FKS+0,5% AB	0	0	0	0	0
EMA 80% 1:2.5	0	4	4	4	4
EMA 80% 1:5	0	3	3,5	4	4
EMA 80% 1:7.5	0	2	2	3,5	3,5
EMA 80% 1:10	0	1	2	2	2
EMA 80% 1:25	0	1	1	0	0
EMA 80% 1:50	0	1	1	0,5	0,5
EMA 80% 1:75	0	1	1	0,5	0
EMA 80% 1:100	0	1	1	0,5	0
EMC 60% 1:2.5	0	2	3	4	4
EMC 60% 1:5	0	1	1	2	2
EMC 60% 1:7.5	0	1	1	2	2
EMC 60% 1:10	0	1	1	2	2
EMC 60% 1:25	0	1	1	0	0
EMC 60% 1:50	0	1	1	0,5	0

EMC 60% 1:75	0	1	1	0	0
EMC 60% 1:100	0	0	0	0	0

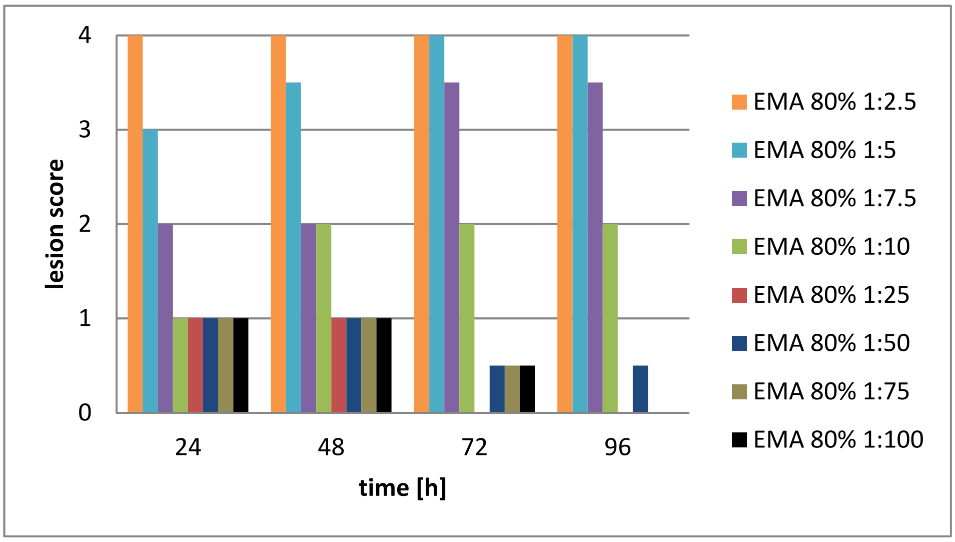

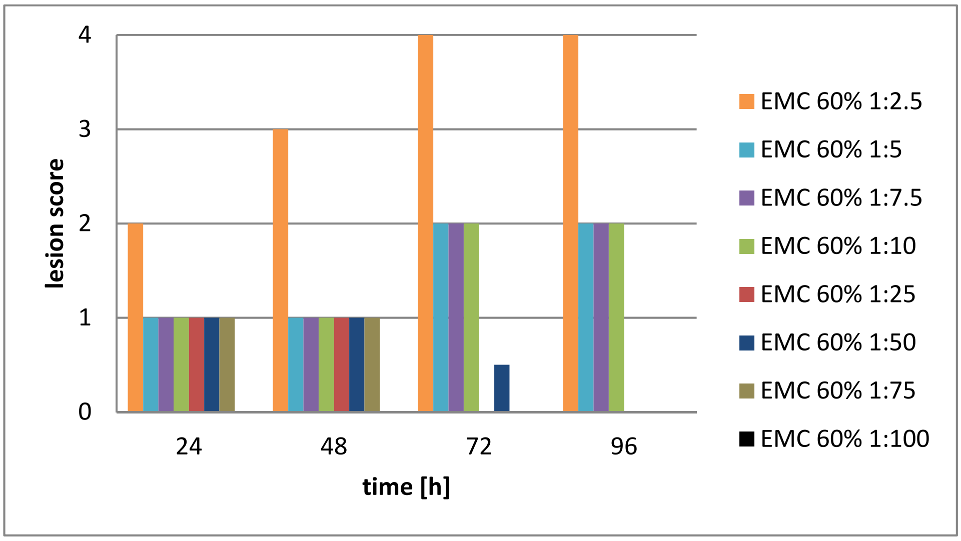

Supplement: Supplementary file 1 [file antibiotics-12-01462-s001.zip › antibiotics-2561092-supplementary.pdf]
